# Evaluating Stress in Dogs Involved in Animal-Assisted Interventions

**DOI:** 10.3390/ani9100833

**Published:** 2019-10-19

**Authors:** Sara Corsetti, Miriam Ferrara, Eugenia Natoli

**Affiliations:** 1 School of Animal Biology, The University of Western Australia, Crawley, Western Australia 6009, Australia; sara.corsetti@research.uwa.edu.au; 2 Cooperativa Sociale Le Mille e Una Notte, Rome 00141, Italy; verdun7843@gmail.com; 3 Canile Sovrazonale, ASL Roma 3, Rome 00148, Italy

**Keywords:** animal-assisted interventions, dog welfare, stress, behaviour

## Abstract

**Simple Summary:**

Dogs are widely involved in animal-assisted interventions (AAIs), but little information is available to determine if AAIs are stressful for dogs. Maintaining the animal wellness is ethically crucial and it is decisive for the success of the AAIs. This study wanted to assess if dogs were stressed during the sessions. Nine dogs, belonging to the A.N.U.C.S.S. (the National Association for the Use of Dogs for Social Aims) association, were observed before, during, and after AAIs with patients—who had mental and/or physical disabilities—to underline any signs of stress. Our results suggested that our dogs were not stressed, as the level of anxious behaviour was low and similar in all three kinds of sessions (before, during, and after sessions).

**Abstract:**

Animal-assisted interventions (AAIs) are co-therapies in which the animal is an integral and active part of the treatment process. Dogs are widely involved in AAI projects, but little data are available to determine if AAI sessions are a source of stress for the dogs. Understanding the emotional state of animals and highlighting any signal of stress is crucial maintaining the wellness of the animals and in enhancing the probability of success of the AAI. The purpose of this study is to assess if dogs present signs of stress during animal assisted therapies sessions. The sample consisted of nine dogs, belonging to the members of the A.N.U.C.S.S. (the National Association for the Use of Dogs for Social Aims) association. Dogs lived with their owners and their health was checked by a vet once a week. Patients involved in the AAI project had disabilities due to mental disorder and/or psychomotor problems. During the therapeutic sessions, patients had to guide the dog along facilitated agility routes and/or perform the activities of cuddling and brushing the dog. When a dog accomplished a task, the patient gave him/her a reward (throwing a ball or a biscuit). Dogs were observed for a total of 174 h, 47 h before, 81 h during, and 46 h after AAI sessions. Each session of observation lasted 10–30 min. The differences of behavioural patterns in the three contexts were analysed by mean of the non-parametric Friedman test. Dogs never showed aggressive and stereotyped behaviour; the level of anxious behaviour was low and similar in all three kinds of sessions. During therapeutic sessions, attention, affiliative behavioural patterns, and sniffing behaviour increased. The highest level of attention of dogs was directed toward their conductor, rather than to the patient and to the other operator present. The results suggest that the amount of work dogs went through was adequate and that dogs did not show behavioural signs of stress.

## 1. Introduction

Animal-assisted interventions (AAIs), formerly called AAA, animal-assisted activities, and AAT, animal-assisted therapies, are co-therapies in which the animal, which has been trained in a specific way, is an integral and active part of the treatment process [1]. The interventions aim to promote physical, emotional, social, or cognitive functions in human beings, such as in cardiopathic or disabled people. Many studies have underlined the benefits for humans from AAIs [2]; they can improve the ability to socialize or communicate, or help with motor rehabilitation (see, for example, [2,3,4,5,6,7,8,9,10]). Furthermore, they can also improve impulse control, enhance self-esteem, and decrease feelings of anger and symptoms of depression and/or anxiety (see, for example, [11,12,13,14,15,16,17,18,19,20,21]).

Dogs are widely involved in AAI projects. Although there is increasing interest in human society in AAIs for their strong benefit on human health, few data are available to assess whether AAI sessions are stressful for dogs (reviewed in [22]). In these situations, animals have a central role in that they have to be constantly monitored to highlight signals of tiredness and/or stress during the therapeutic sessions.

Stress is a state characterized by a specific syndrome (increased activity of the sympathetic nervous system, high catecholamine production, hypertension, etc.) that can be triggered by various factors—the stressors (infections, wounds, burns, but also anger, overwork, etc.) [23]; therefore, any stimulus that provokes, for example, pain, fear, heat, cold, blood-loss, environmental contaminants, pathogenic microbes, excessive tiredness, or social tensions. Reaction to stress refers to non-specific changes in an organism caused by an emotional or physical disorder.

Therefore, in situations that the animal perceives as undesirable, there can be behavioural and/or physiological manifestations that reveal a status of stress.

Dogs can experience stress in many situations, including AAI sessions (e.g., [24,25,26]), and their reaction can range from physiological to immunological, as well as behavioural changes (see [27,28,29,30]). Understanding the emotional state of dogs and highlighting any signal of fatigue and/or stress is crucial in order to maintain the wellbeing of the animals and to enhance the probability of success of animal-assisted interventions. 

The observation of behaviour is a non-invasive method of gathering information on the animal, and for this reason the quantitative measurement of the different behavioural patterns (including those that report a state of stress) is potentially of great importance, as the results are minimally influenced by the sampling techniques.

By means of behavioural analysis, the aim of this study was to evaluate whether dogs involved as co-therapists in animal-assisted interventions showed behavioural signs revealing a state of fatigue and/or stress. The behavioural patterns useful for this purpose have been identified on the basis of the available literature on behavioural indicator of stress (see, for example, [29,30,31,32]).

The nine dogs involved in the study were carefully selected by the A.N.U.C.S.S. association (the National Association for the Use of Dogs for Social Aims) according to the ministerial Italian guidelines for animal-assisted interventions (AAI); furthermore, the dogs had experience as co-therapists in AAI.

We are aware that the dog sample size was small, however, to carry out behavioural observations with ethological traditional methods requires a stable and lasting presence (many hours) in the medical setting, which can disturb the therapy and, at the same time, pose some problems in preserving the privacy. Consequently, it is extremely difficult to obtain the authorisations necessary to attend the therapy sessions. Moreover, the careful selection of dogs operated by the association inevitably reduced the number of animals. 

## 2. Materials and Methods 

### 2.1. Animals

The sample consisted of nine dogs—six females (one spayed) and three males (one neutered)—aged between 1 and 7 years old. Seven dogs were purebred (Golden Retriever) and two dogs were mixed breed (Golden Retriever × Labrador and unknown mixed breed) (Table 1).

All dogs belonged to the members of the A.N.U.C.S.S. association (the National Association for the Use of Dogs for Social Aims), a non-profit organisation that has operated since 1998 in the field of AAIs. Dogs lived with the owners (which were the conductors at the same time) and their health was checked by a vet once a week. Eight owners/conductors were involved in the study.

### 2.2. Dogs’ Selection

According to the Ministerial Italian Guidelines for AAI, animals accepted as pet partners must have passed an assessment regarding health control, skills, and aptitude. Concerning skills, the dog must show that it is able to accept a stranger amicably, to sit down quietly, to be caressed, to walk in a group of people, to respond to basic commands given by the conductor (such as “sit”, “stay”, “come”), and to remain calm in front of various distractions. Aptitude is evaluated by a simulation during which the animal must accept, for example, to be manipulated by a stranger, to be caressed by an agitated person who has a high tone of voice, to be hugged, to receive a sudden hit, to be surrounded and caressed by several people at the same time and, despite all this, to be sociable.

All the dogs involved in this study passed the prescribed tests and had been accepted as pet partners.

### 2.3. Medical Set

Patients involved in the AAI project had disabilities due to mental disorders and/or psychomotor problems, for example, Rett syndrome, autism spectrum disorder (ASD), and attention deficit hyperactivity disorder (ADHD). AAI sessions consisted of (1) “agility” routes—the patient had to guide the dog along these facilitated agility routes, overcoming small obstacles, doing slalom, and so forth. When a dog accomplished a task, the patient gave him a reward (throwing a ball or a biscuit); (2) activity of cuddling and brushing the dog; (3) for patients in a wheelchair, the task consisted of throwing a ball or a biscuit to the dog.

### 2.4. Observations and Location

Dogs were observed by a unique observer for a total of 174 h—47 h before, 81 h during, and 46 h after AAI sessions. Given that the number of observation hours for each dog was not the same, data were normalized by dividing number of events by individual time of observation.

Each session of observation lasted between 10 and 30 min. Dogs took part in a maximum of four sessions a day, which contained an interval of at least 2 h between each session. In each therapeutic session, one patient was involved at once, together with the conductor of the dog; furthermore, one sanitary operator (that could be a nurse, physiotherapist or a psychologist) and the observer of dog behaviour were present.

Behavioural observations were carried on following the release of the necessary authorisations from the A.N.U.C.S.S. association, the hosting sanitary structure, and the relatives of patients involved.

Data were collected by behavioural observations before, during, and after therapeutic sessions. Before and after the sessions, dogs were observed in places different from the medical setting, where dogs were accustomed to wait before being involved in the AAI sessions or before going back to owners’ houses. The ethogram utilised (Supplementary Materials Appendix A) consisted of 53 behavioural patterns gathered into 6 categories: dominance, aggressiveness, submission, affiliative behaviour, anxiety, and attention. Data were collected using the focal animal sampling method by means of ‘all occurrences’ recording method [33]. For the description of each behavioural patterns, see Supplementary Materials Appendix A.

Interference due to the presence of the observer was minimized because she collected the behavioural data standing on the sidelines, motionless and in silence, as is usual in all behavioural studies.

There were three different locations in which dogs worked. The first was the training campsite of the A.N.U.C.S.S. association, where the sessions were carried out outdoors in an enclosure, and were equipped for agility paths; the second location was in the psycho-motor rehabilitation centre of Villa Alba, where therapies were carried out indoors in gyms or rooms, and outdoors in courtyards; the third location was in the Grassi Hospital in Ostia (Rome), in the games room, indoors. All behavioural observations were carried on in the context in which the dogs were familiar, in order to avoid a change in the routine that could have been a source of stress that would be capable of influencing the results—the dog being an animal extremely sensitive to routine changes [26].

The owners/conductors were asked to fill a questionnaire on their dogs involved in this study. The questionnaire was the Canine Behavioural Assessment and Research Questionnaire (C-BARQ). This questionnaire is a standardized, behavioural evaluation tool for dog owners/guardians, handlers, and professionals that provides a set of numerical scores for 14 different categories of dog behaviour (Table 2). It was developed and validated by Hsu and Serpell [34].

### 2.5. Statistical Analysis

The differences of behavioural patterns in the three contexts were analysed by means of the non-parametric Friedman test. This test is utilised to detect differences in treatments across multiple test attempts.

The personalities of the dogs were determined by principal component analysis (PCA) utilising the numerical scores provided for the 14 different categories of the C-BARQ. The PCA factors were named on the basis of variables showing correlations with them (significance set at 0.60); furthermore, Spearman rank correlation coefficient was applied to assess the relationship between a dog’s individual score for each factor and the level of anxiety showed by the dog during the therapeutic sessions.

All statistical analyses were carried out using IBM SPSS Statistics release 25 (IBM Corp. Released 2017 Armonk, NY, USA).

### 2.6. Ethics Statements

We did not need institutional or governmental permission to carry on the study because it was an observational study. Neither anaesthesia nor euthanasia, or any kind of animal sacrifice, was part of the study.

## 3. Results

### 3.1. Personality of Dogs

The first four factors in the PCA explain 86.44% of the total variance of the data. For the factors, we considered relevant any correlation of 0.60 or above for the variable loading (Table 3, highlighted in yellow).

The individual dog values for each factor highlighted by the PCA allowed us to describe the personality of the dog (Table 1). The first factor (F1) was highly correlated with measures of “trainability” versus measures of “aggression, excitability, energy, fear of strange people, problems related to attachment/separation, problems related to manipulation”. Thus, dogs with a high negative score for the first factor were dogs with a high capacity to be trained and low aptitude to (i) show intra- and inter-specific aggressive behaviour, (ii) be frightened by unknown people, (iii) show separation anxiety, (iv) maintain close proximity to the owner and display agitation when the owner gives attention to third parties, (v) chase other animals, (vi) display strong reactions to potentially exciting or arousing events, (vi) be frightened by close contact with people, and (vii) be very energetic.

The second factor (F2) was highly correlated with the variable “intra-specific aggressive behaviour towards familiar dogs” and, thus, dogs with a high positive score for the second factor were dogs that basically showed rivalry towards familiar dogs.

On the contrary, because the third factor (F3) was highly correlated with the variable “dog-directed fear” and, on the other direction, with “owner-directed aggression”, dogs with a high positive score for the third factor were dogs that basically showed fear towards unfamiliar dogs and low aggressive behaviour towards the owner.

Finally, the fourth factor (F4) was highly correlated with the variable “non-social fear”, and dogs with a high positive score for the fourth factor were dogs that showed fear or wary responses to sudden or loud noises, traffic, and unfamiliar objects and situations.

On the base of dog individual score (their order in brackets) for each factor, it was possible to outline their personality (Table 4).

A consideration is due—C-BARQ questionnaire variables utilise the word “aggression” and, thus, we were obliged to define “aggressive” the dogs that had high individual scores for that variable. However, dogs were evaluated by the PCA in relation to themselves. Thus, one who was defined as aggressive was probably not very aggressive in general but was the most aggressive of the group. None of the dogs involved in the AAI were actually aggressive, otherwise they would not have passed the prescribed tests and would not have been accepted as pet partners.

### 3.2. Dog Behaviour During the Therapeutic Sessions 

During the therapeutic sessions, the dogs never showed any kind of aggressive behaviour; the same was true for stereotyped behaviour—they never showed compulsive or repetitive behaviour such as licking objects, compulsive autogrooming, or pacing.

**Figure 1:**
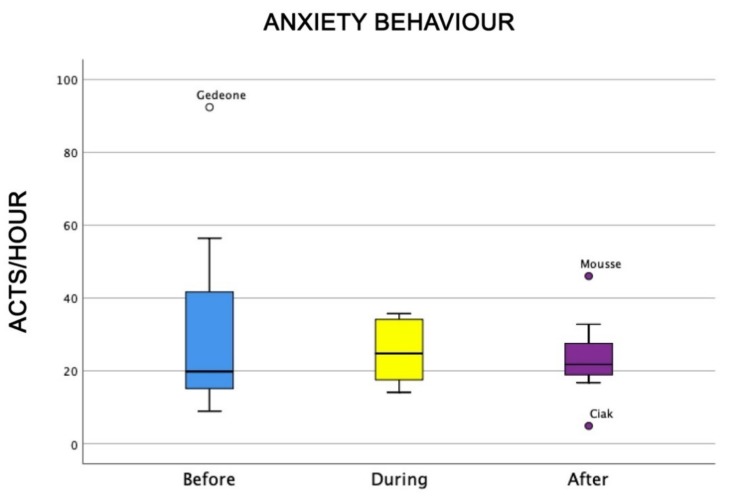
Behavioural patterns related to dogs’ level of anxiety towards humans before, during, and after animal-assisted intervention (AAI) sessions. Non-parametric data are represented as medians (horizontal bar in the box) and the box indicates the interquartile range of 50% of the data. Whiskers extend to the smallest and largest values and exclude outliers (dot on top).

The level of anxious behaviour was similar in all three kinds of sessions (anxiety: X_2_ = 0.25, df = 2, NS) (Figure 1).

**Figure 2:**
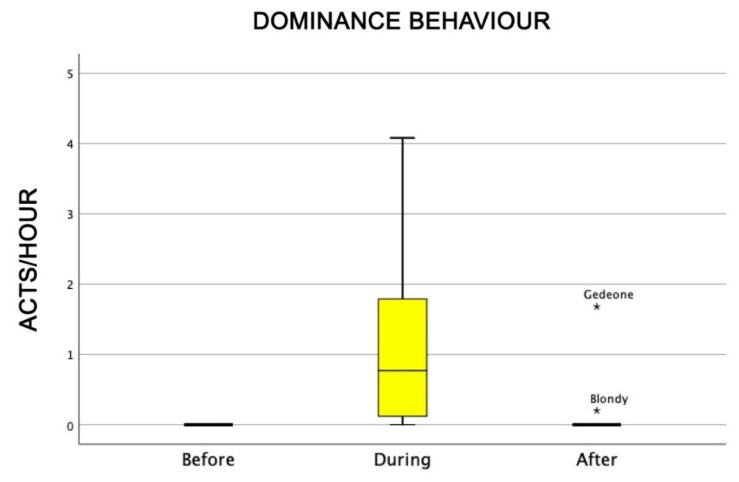
Behavioural patterns related to dogs’ dominance behaviour before, during, and after AAI sessions. Non-parametric data are represented as medians (horizontal bar in the box) and the box indicates the interquartile range of 50% of the data. Whiskers extend to the smallest and largest values and exclude outliers (dot on top).

**Figure 3:**
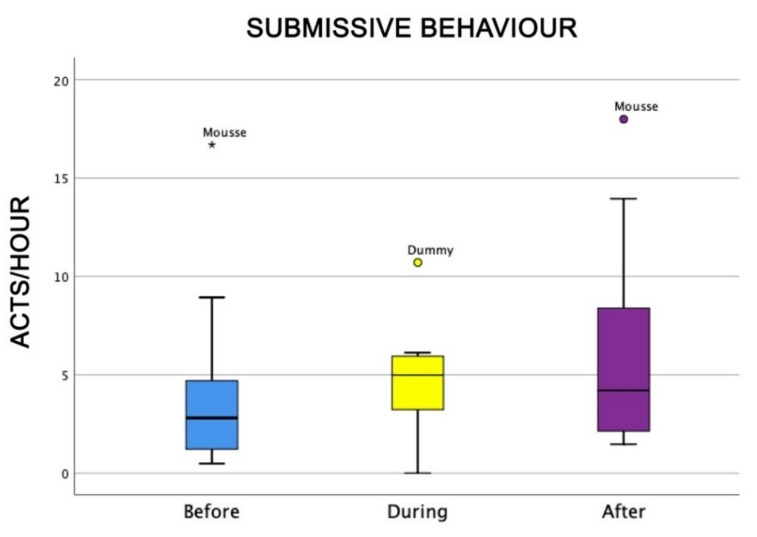
Behavioural patterns related to dogs’ submissive behaviour before, during, and after AAI sessions. Non-parametric data are represented as medians (horizontal bar in the box) and the box indicates the interquartile range of 50% of the data. Whiskers extend to the smallest and largest values and exclude outliers (dot on top).

Dominance behaviour (that was not linked to aggressive behaviour) was higher during AAI sessions than before and after the sessions themselves (Friedman test: χ^2^ = 9.5, df = 2, *p* = 0.008) (Figure 2), whereas submissive behaviour did not change significantly among the sessions (Friedman test: χ^2^ = 4.7, df = 2, NS) (Figure 3).

**Figure 4:**
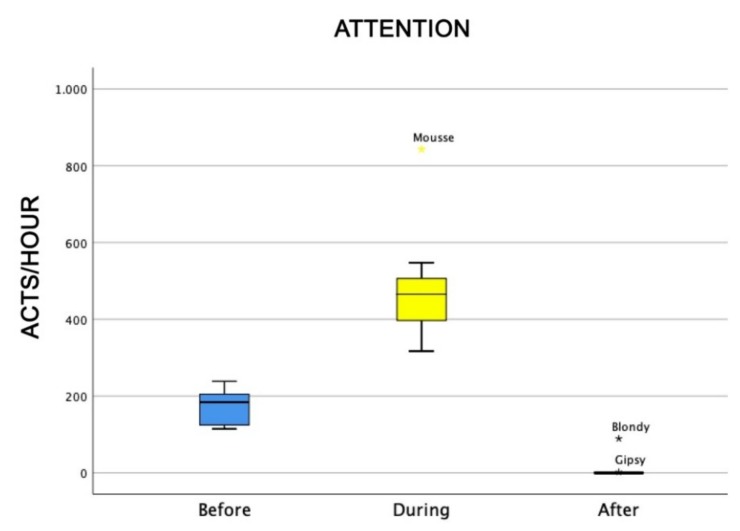
Behavioural patterns related to dogs’ level of attention before, during, and after AAI sessions. Non-parametric data are represented as medians (horizontal bar in the box) and the box indicates the interquartile range of 50% of the data. Whiskers extend to the smallest and largest values and exclude outliers (dot on top).

**Figure 5:**
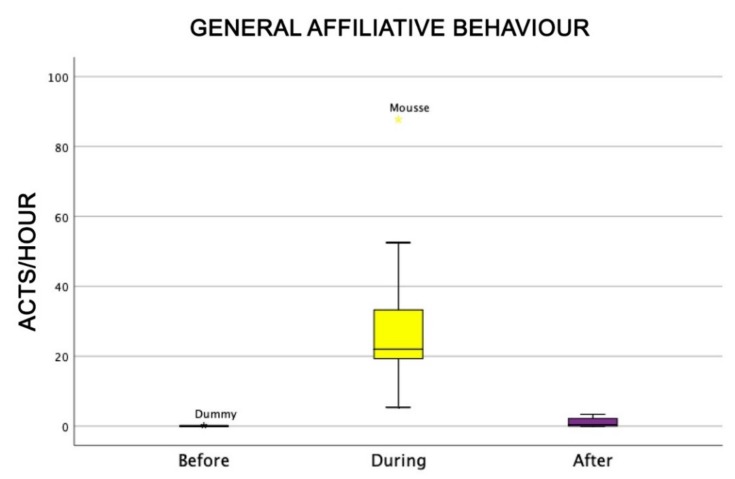
Behavioural patterns related to dogs’ general affiliative behaviour before, during, and after AAI sessions. Non parametric data are represented as medians (horizontal bar in the box) and the box indicates the interquartile range of 50% of the data. Whiskers extend to the smallest and largest values and exclude outliers (dot on top).

**Figure 6:**
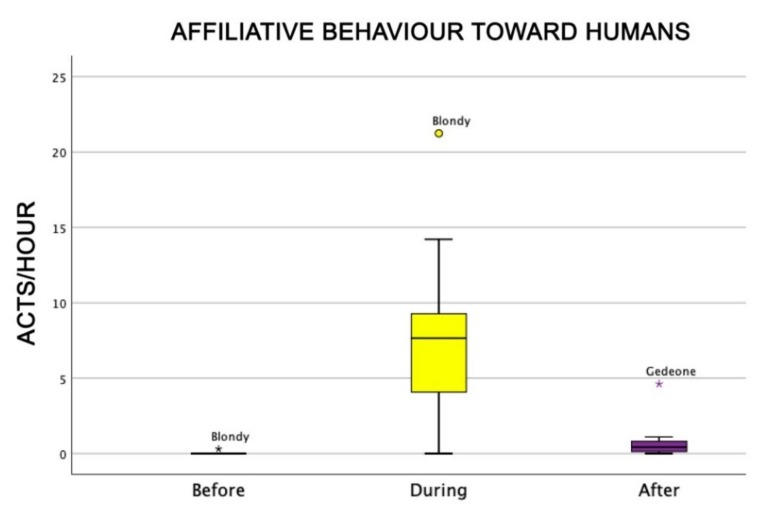
Behavioural patterns related to dogs’ affiliative behaviour towards humans before, during, and after AAI sessions. Non-parametric data are represented as medians (horizontal bar in the box) and the box indicates the interquartile range of 50% of the data. Whiskers extend to the smallest and largest values and exclude outliers (dot on top).

**Figure 7:**
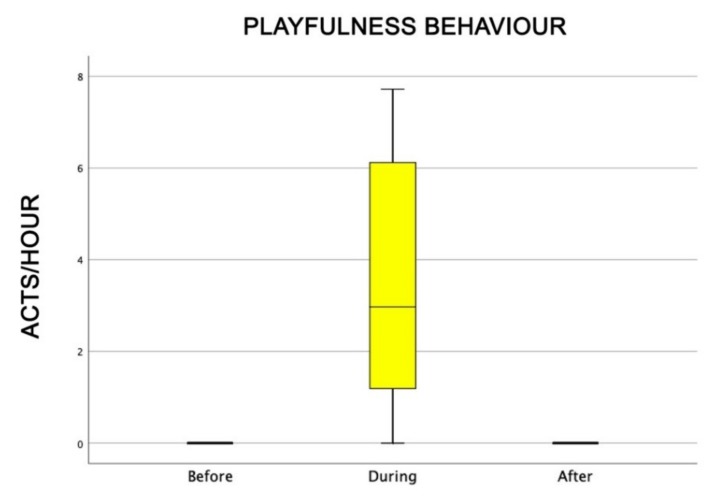
Behavioural patterns related to dogs’ playful behaviour towards humans before, during, and after AAI sessions. Non-parametric data are represented as medians (horizontal bar in the box) and the box indicates the interquartile range of 50% of the data. Whiskers extend to the smallest and largest values and exclude outliers (dot on top).

**Figure 8:**
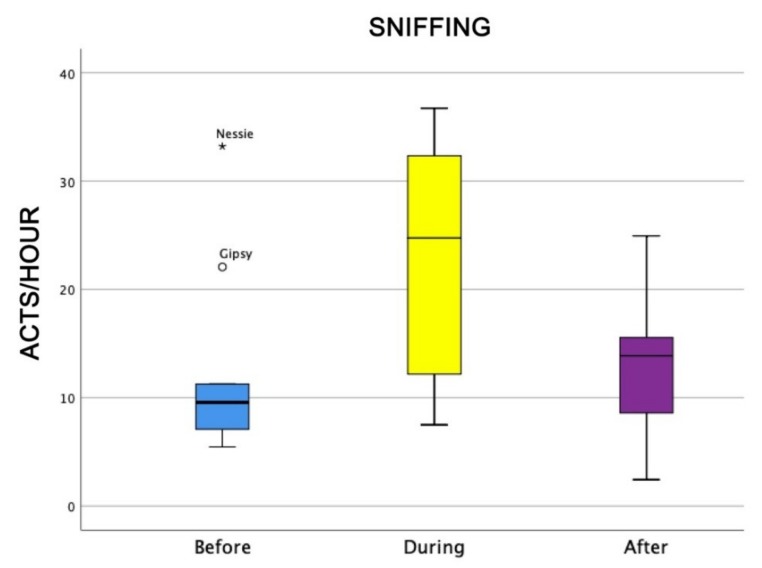
Behavioural patterns related to dogs’ level of sniffing before, during, and after AAI sessions. Non-parametric data are represented as medians (horizontal bar in the box) and the box indicates the interquartile range of 50% of the data. Whiskers extend to the smallest and largest values and exclude outliers (dot on top).

**Figure 9:**
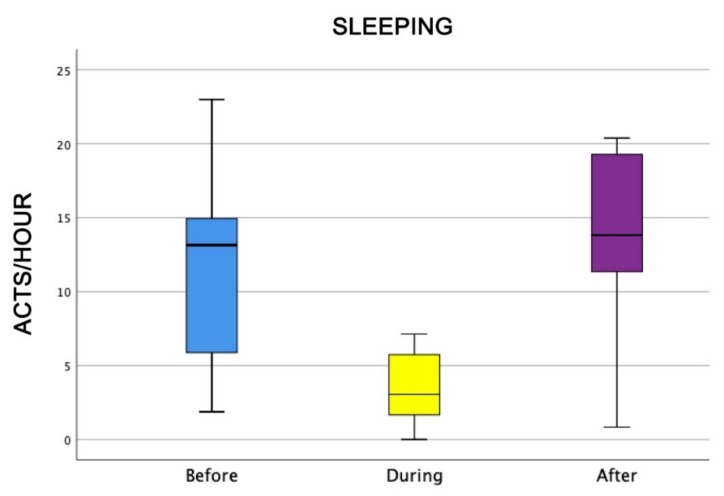
Number of times dogs had been sleeping before, during, and after AAI sessions. Non-parametric data are represented as medians (horizontal bar in the box) and the box indicates the interquartile range of 50% of the data. Whiskers extend to the smallest and largest values and exclude outliers (dot on top).

Also, the level of attention of dogs was higher during AAI sessions than before and after (Friedman test: χ^2^ = 18.0, df = 2, *p* = 0.0001) (Figure 4), as well as dogs’ general affiliative behaviour (Friedman test: χ^2^ = 16.9, df = 2, *p* = 0,0001) (Figure 5), dogs’ affiliative behaviour towards humans (Friedman test: χ^2^ = 15.5, df = 2, *p* = 0.0001) (Figure 6), playful behaviour (Friedman test: χ^2^ = 16, df = 2, *p* = 0.0001) (Figure 7), and sniffing behaviour (Friedman test: χ^2^ = 6.9, df = 2, *p* = 0.03) (Figure 8). On the contrary, as one might expect, dogs spent less time sleeping during the therapeutic sessions (Friedman test: χ^2^ = 13.6, df = 2, *p* = 0.001) (Figure 9).

The affiliative behaviour patterns towards humans were significantly higher during sessions (Friedman test: χ^2^ = 15.5, df = 2, *p* = 0.0001).

**Figure 10:**
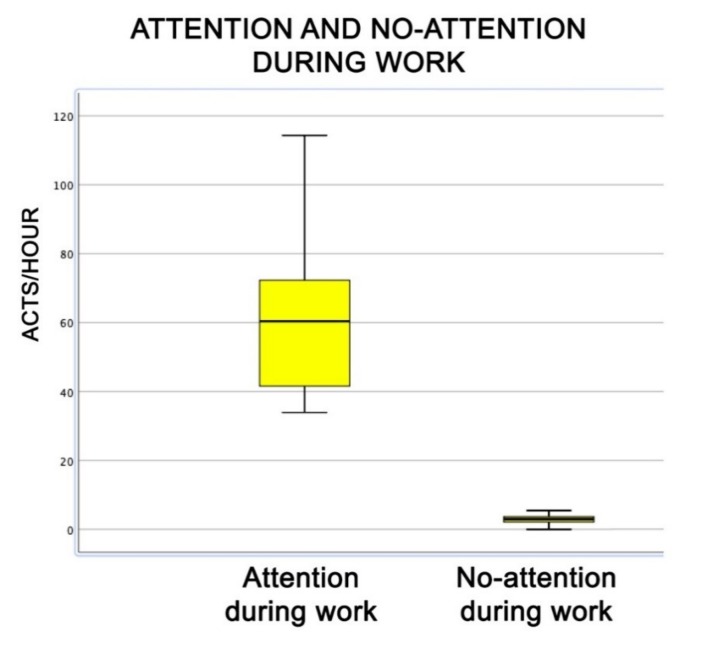
Behavioural patterns related to dogs’ level of attention/no attention to work during the AAI sessions. Non-parametric data are represented as medians (horizontal bar in the box) and the box indicates the interquartile range of 50% of the data. Whiskers extend to the smallest and largest values and exclude outliers (dot on top).

**Figure 11:**
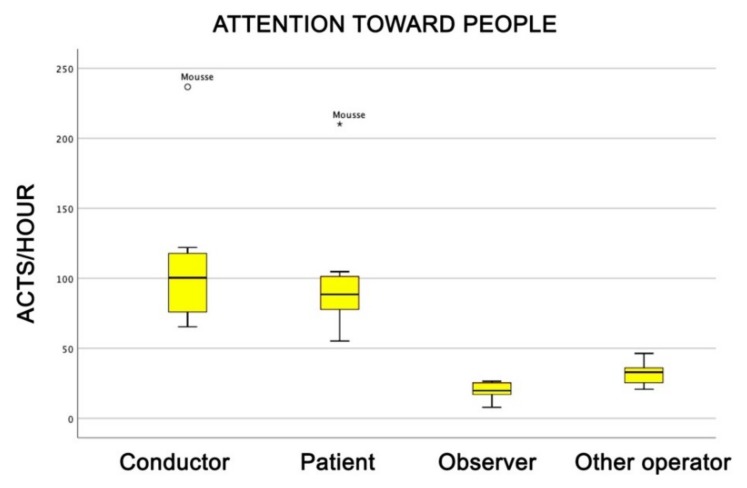
Number of gazes per hour shown by dogs during the AAI sessions. Non-parametric data are represented as medians (horizontal bar in the box) and the box indicates the interquartile range of 50% of the data. Whiskers extend to the smallest and largest values and exclude outliers (dot on top).

During the therapeutic sessions, dogs rarely showed a low level of interest in what they were involved in (Figure 10) and, even more interesting, their attention, measured in number of gazes, was considerably higher towards the conductor than towards the patient or towards other people (Friedman test, attention: χ^2^ = 25.1; df = 3; *p* = 0.0001) (Figure 11).

**Figure 12:**
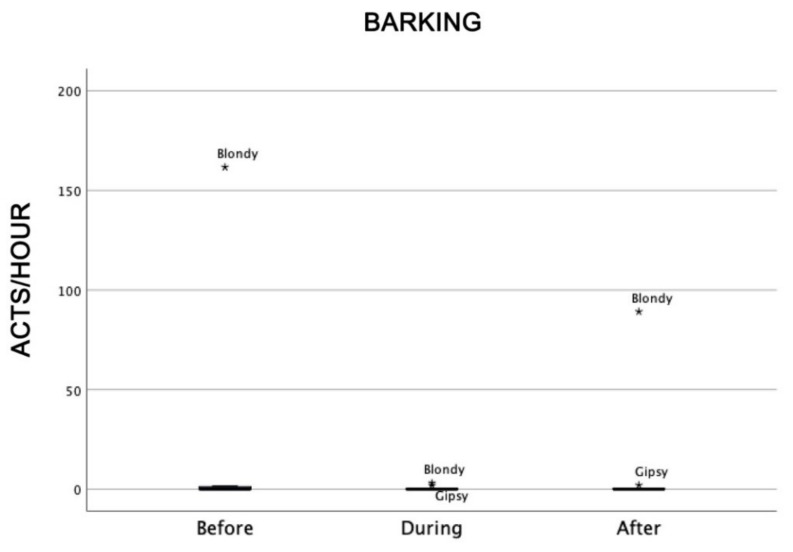
Barking of dogs before, during, and after AAI sessions. Non-parametric data are represented as medians (horizontal bar in the box) and the box indicates the interquartile range of 50% of the data. Whiskers extend to the smallest and largest values and exclude outliers (dot on top).

A low level of barking was registered for all but one dog; Blondy barked considerably, but mainly before and after the therapeutic sessions (Figure 12).

There was no correlation between dog individual score of each factor of the PCA and the level of anxiety shown during the therapeutic session (Spearman correlation test, two tailed; anxiety and factor 1: rho = 0.20, *N* = 9, *p* = 0.47; anxiety and factor 2: rho = 0.83, *N* = 9, *p* = 0.08; anxiety and factor 3: rho = 0.78, *N* = 9, *p* = 0.11; anxiety and factor 4: rho = 0.10, *N* = 9, *p* = 0.59).

## 4. Discussion

This study aimed to examine whether dogs involved in animal-assisted therapies, in the context described here, were stressed. Despite the small dogs sample size, a common limitation of these kinds of studies (reviewed in [22]), our results seem quite clear—the amount of work the dogs went through was adequate, the relationship with the conductor/owner was correct (the dog looked at the conductor to understand what to do), and dogs did not show visible signs of fatigue and/or stress.

The PCA allowed us to outline the dogs’ personality (see Table 1 for more information). This represents an important premise for this study—as one might expect, dogs were characterized by different personalities. Nevertheless, during the therapeutic sessions, the dogs did not show different levels of anxiety that, of course, might have been due to their different personalities (no difference in displacing activities that indicate a moderate level of anxiety [23,35,36]). Furthermore, dogs neither showed stereotyped behaviours, that are indicators of pathological behaviour due to a discomfort status [31,32,37,38], nor aggressive behaviour that would have shown the dogs’ discomfort/fear/intolerance. Though with a moderate frequency, during the therapeutic sessions anxiety behaviour was shown, probably because very often the patients—that mostly had both cognitive, psychological, and physical disabilities—had problems in giving the rewards (a biscuit or a ball) to the dogs. The patient hesitation increased the frequency of some behavioural patterns in dogs (especially the panting and the licking of the muzzle). Without this hesitation, the level of displacement activities would have been even lower.

The fact that the relationship between the dogs and the conductor/owner was correct was supported not only by the fact that—despite their different personalities—when working near their own owner, dogs were not anxious, and this was also supported by several behavioural indicators. Firstly, during the therapeutic sessions, dogs behaved as though they were confident; they showed signs of dominance (such as tail held high and pricked-up ears, but not related to aggressive behaviour) but did not show more submissive behaviour in the presence of the conductor compared with in the absence of him/her. These results seem to suggest that the dogs did not live the AAI as a constriction. Secondly, dogs showed a high level of attention, sniffing, and affiliative behaviour, including playful behaviour, during the therapeutic sessions. Although this result might be predictable because during the therapeutic session dogs had more opportunity to interact with humans, nevertheless the interactions with humans could have been either positive or negative. In actuality, dogs might have been unwilling to interact, bored, or frightened. Thus, it is worthwhile underlining the higher frequency of affiliative behaviour during the therapeutic sessions because it is well known that amicable behaviour denounces a state of relaxation without aggression [39]. Finally, it is important to recognise that dogs live in an olfactory world, and an inadequate behaviour of sniffing could have been due to a malaise situation [39].

The results seem to suggest that the dogs were willing to participate as co-therapist, and that this enhanced the probability of success of the therapy; in fact, for the success of any AAI, it is important that dogs wish to take part in sessions without any coercion. In actuality, playful behaviour was displayed only during the therapeutic sessions, never before or after them. 

As mentioned in the method section, we decided not to modify dogs’ routine before and after the therapeutic sections because, as Rooney et al. [26,32] reported, the changes of daily habits in dog routine can be an important source of stress in itself. This means that we decided to maintain very different sets before and after the therapeutic sessions, during which behavioural data were collected because, in the balance costs/benefits, the variability of the contests biased the behaviour less than the disruption of the routine.

As already discussed, the attention showed by dogs during the therapeutic sessions was higher than before and after the sessions. This supports the impression that the dogs were active and wanted to take part in the sessions, in which they were very participative. Dogs looked at the patient very often. This is predictable, because the patient held in one hand the reward for the dog (a biscuit or a ball) and, consequently, dogs were stimulated to look at them. However, more interestingly, more often the dogs pointed their gazes towards the conductor—that being the owner. This supports what was stated above, that is, that the owner–dog relationship was strong and trustful. In fact, it has been shown that dogs pay more attention to the actions of their owners than to the actions of other familiar humans, indicating that in these animals, attention is enhanced by affiliation [40], but also by rank. In the mixed group of humans–dogs, the place of the alpha male is often occupied by the owner and the highest level of attention is dedicated to him/her. The phenomenon goes under the name of “the social structure of attention”. The first time it was described in a social group of long-tailed macaques (*Macaca fascicularis*), where it was found a strong positive correlation between the dominance rank and the frequency and duration at which an animal was visually followed by other members of the group. In other words, the dominant animal was the focus of the attention of all other individuals [41]. This tendency has been found also in social groups of free-roaming dogs [42] and in wolves [43,44].

Furthermore, in the last decades, a line of research has been developed on visual communication between human beings and dogs. The findings have led to an amount of evidence showing that the behaviour of dogs is strongly influenced by human facial expressions and gestures [45,46,47,48]. As Miklosi et al. [49] pointed out “…the readiness of dogs to look at the human face has led to complex forms of dog–human communication”. In particular, it has been found that when a dog cannot solve a task, it looks back toward the conductor for help [50]. It is assumed that dogs base their decisions on the indications given by a known human rather than by a stranger (see, for example, [51]).

Another form of communication in dogs is barking, which can be due to different causes, such as aggressiveness, separation anxiety, or attention request [39,52,53]. We registered a low level of barking before, during, and after the AAI sessions. Only one dog (Blondy) barked consistently before and after the therapeutic sessions because she probably wanted to catch the attention of the conductor in order to be involved in the therapeutic sessions. Frequent barking is usually a problem for both dogs and humans, and various possibilities have to be considered to hypothesize the causes of excessive barking. Interestingly, in this study, the dog that barked more than the others before and after, but not during, the therapeutic sessions had a very strong personality (see Table 1), and that is why we suggested that she was trying to catch the attention of the conductor because she liked working. This result further supports the hypothesis that there was a positive emotional involvement of dogs in working, which is crucial for the success of the therapy.

Finally, dogs slept more before and after AAI sessions, even if they snoozed also during sessions, when owners and patients caressed and brushed them. This result give us an idea about the relaxing status of individuals—they were calm enough to snooze during sessions, even if in strict contact with patients. At the same time, they showed a considerably high level of attention because low frequency of snoozing and high frequency of sniffing confirms also that they were active.

In the last decade, the idea is increasingly widespread that, in order to assess the welfare level of an animal, it is not necessary to consider only the absence of negative affective states but also the presence of positive affective states [54].

Positive effects or emotional states may include pleasure, comfort, contentment, curiosity, and playfulness, which the majority of the dogs showed during the therapeutic sessions in this study.

## 5. Conclusions

The results presented in this paper suggest that the dogs involved in this study, although behaving like normal dogs, showing many behaviours that fall into the normal repertoire of the species, meeting the requirements described in the Materials and Methods section. In fact, they showed the adequate skills and aptitude to be involved in AAI. During the therapeutic sessions, they did not show behavioural signs of stress, probably due to the following causes: (i) the AAI sessions lasted never more than 30 min, (ii) the dogs had proper rest between the therapeutic sessions, (iii) each dog worked for a maximum of four sessions per day, and (iv) there was a correct trustful relationship between dogs and conductors/owners.

Furthermore, these results emphasize that it is not correct to do AAI with inadequately prepared dogs not led by the coadjutor, as during work their personality would not be mediated by a correct relationship.

## Figures and Tables

**Table 1:** The sample of dogs involved in this study. For each individual, the age, sex and sexual status, breed, and personality is indicated.

Dog	Age	Sex	Purebred/Mixed Breed	Personality
Blondy	5 years, 6 months	Female	Golden Retriever purebred	Easily trainable, not very aggressive towards dogs and strangers but quite bold towards known dogs, not aggressive towards the owner, not inclined to predatory behavior, not afraid of strangers, little separation anxiety, she is not intimidated by potentially painful procedures such as nail trimming and visiting the veterinarian, does not often seek the attention of humans, not very excitable, not particularly “always on the move”. A true leader.
Ciak	6 years	Male	Golden Retriever purebred	Quite trainable, not very aggressive towards strangers (people) or unknown and known dogs, but with a moderate tendency to be bold towards the owner; not prone to predatory behavior, not afraid of strangers but avoids confrontation with unknown dogs, not particularly suffering from separation anxiety. In general, he is fearful but, on the contrary, he is intimidated very little by potentially painful procedures such as nail trimming and visits to the veterinarian; he is not easily excitable, does not require much attention, and is not particularly “always on the move”.
Dummy	7 years	Male, neutered	Golden Retrieverx Labrador mixed breed	He responds little to training; not very self-confident towards strangers and is rather wary of unknown dogs; slightly insecure towards those known and rather “stubborn” towards the owner; he is prone to predatory behaviour, is afraid of strangers and, because he tolerates little frustration, suffers moderately from separation anxiety; he is scary and is intimidated by potentially painful procedures such as nail trimming and visits to the veterinarian; he is excitable, seeks the attention of humans, and is dynamic.
Gedeone	3 years	Male	Golden Retriever purebred	Good trainability, he is quite aggressive towards strangers (of whom he is afraid) and towards unknown dogs, but not very aggressive towards those known (of whom he is not particularly afraid); he is rather bold towards the owner; he is prone to predatory behaviour, suffers from separation anxiety, is fearful and is quite intimidated by potentially painful procedures such as nail trimming and visits to the vet, is excitable, requires a lot of attention from humans, and is quite dynamic.
Gipsy	11 years, 10 months	Female, spayed	Unknown mixed breed	Fair trainability, on average aggressive towards strangers (of whom she has a certain fear) and towards unknown dogs, but never aggressive towards those known with whom she tends to avoid confrontation; she is moderately bold towards the owner; she is not particularly prone to predatory behaviour, and suffers moderately from separation anxiety; she is not a generally fearful dog and is not intimidated by potentially painful procedures such as nail trimming and visits to the veterinarian; she is averagely excitable, requires average attention from humans, and is quite dynamic.
Indi	1 year, 4 months	Female	Golden Retriever purebred	Poor trainability because she was not very motivated, she did not really want to collaborate; on average aggressive towards strangers (of which she has a certain fear) and towards unknown dogs, but never aggressive towards those known, as she has a subordinate role; she is on average “stubborn” towards the owner; prone to predatory behaviour, suffering in a controlled manner from separation anxiety, is a moderately fearful dog, and does not like potentially painful procedures such as nail trimming and visits to the veterinarian; she is averagely excitable, requires average attention from humans, and is quite dynamic.
Minnie	1 year	Female	Golden Retriever purebred	Good trainability, little aggressive towards dogs and strangers but quite bold towards known dogs; lack of aggressiveness towards the owner; not prone to predatory behaviour, no fear of strangers, but afraid of other dogs; shows little separation anxiety, is not intimidated by potentially painful procedures such as nail trimming and visiting the veterinarian; she is a little excitable dog, she does not always try to be the centre of attention of humans, and she is not particularly active.
Mousse	1 year	Female	Golden Retriever purebred	Not particularly easy to train because of her tendency to be independent and bold towards unknown and known dogs (towards which she has a total absence of fear), and towards strangers; she is “stubborn” also with the owner—it was necessary to fight a little to impose the point on her; very prone to predatory behaviour, showing a discreet fear towards strangers; shows a certain level of separation anxiety, is intimidated by potentially painful procedures such as nail trimming and visiting the vet; she is rather excitable, active, and she often seeks the attention of humans.
Nessie	1 year	Female	Golden Retriever purebred	Somewhat trainable, wary of unknown and known dogs (towards which he shows submissive behaviour), and towards strangers; absence of aggression towards the owner; prone to predatory behaviour, he is afraid of strangers; manifests separation anxiety, is intimidated by potentially painful procedures such as nail trimming and visiting the veterinarian; he is rather excitable, active, and he often seeks the attention of humans.

**Table 2:** The 14 different categories of dog behaviour included in the C-BARQ questionnaire.

C-BARQ Questionnaire’s Categories	
Stranger-directed aggression	Threatening or hostile responses to strangers approaching or invading the dog’s or owner’s personal space, territory, or home range.
Owner-directed aggression	Threatening or hostile responses to the owner or other members of the household when challenged, manhandled, stared at, stepped over, or when approached while in possession of food or objects.
Dog-directed aggression	Threatening or hostile responses when approached by unfamiliar dogs.
Dog rivalry	Threatening or hostile responses to other familiar dogs in the same household.
Stranger-directed fear	Fearful or wary responses when approached by strangers.
Non-social fear	Fearful or wary responses to sudden or loud noises, traffic, and unfamiliar objects and situations.
Non-social fear	Fearful or wary responses to sudden or loud noises, traffic, and unfamiliar objects and situations.
Dog-directed fear	Fearful or wary responses when approached by unfamiliar dogs.
Separation-related behaviour	Vocalizing and/or destructiveness when separated from the owner, often accompanied or preceded by behavioural and autonomic signs of anxiety, including restlessness, loss of appetite, trembling, and excessive salivation.
Attachment and attention-seeking	Maintaining close proximity to the owner or other members of the household, soliciting affection or attention, and displaying agitation when the owner gives attention to third parties.
Trainability	Willingness to attend to the owner, obey simple commands, learn quickly, fetch objects, respond positively to correction, and ignore distracting stimuli.
Chasing	Chasing cats, birds, and/or other small animals, given the opportunity.
Excitability	Displaying strong reactions to potentially exciting or arousing events, such as going for walks or car trips, doorbells, arrival of visitors, and the owner arriving home; has difficulty settling down after such events.
Touch sensitivity	Fearful or wary responses to potentially painful procedures, including bathing, grooming, nail-clipping, and veterinary examinations.
Energy level	Energetic, “always on the go”, and/or playful.

**Table 3:** Results from the principal component analysis (PCA): factors and relevant correlations with behavioural categories (highlighted in yellow).

	Factors			
	F1	F2	F3	F4
**Stranger-directed aggression**	0.686	0.621	0.065	0.074
**Owner-directed aggression**	0.426	0.549	−0.606	−0.243
**Dog-directed aggression**	0.746	0.465	−0.404	0.197
**Familiar dog aggression (dog rivalry)**	0.105	0.871	0.273	−0.326
**Stranger-directed fear**	0.658	−0.070	0.503	0.439
**Non-social fear**	−0.051	0.499	0.272	0.649
**Dog-directed fear**	−0.189	0.418	0.702	−0.446
**Separation-related problems**	0.933	0.040	−0.049	−0.160
**Attachment/attention-seeking**	0.797	−0.331	0.441	−0.165
**Trainability**	−0.740	0.241	−0.119	0.169
**Chasing**	0.865	0.089	−0.045	0.186
**Excitability**	0.840	−0.381	−0.064	−0.101
**Touch sensitivity**	0.922	−0.136	0.052	0.125
**Energy**	0.845	−0.205	−0.056	−0.221

**Table 4:** Dog individual score (their order in brackets) for each factor.

Dogs	Individual Scores Per Factor			
	F1	F2	F3	F4
**Blondy**	−0.83 (9)	0.49 (7)	−0.43 (4)	0.19 (5)
**Ciak**	−0.56 (7)	−0.30 (4)	0.15 (7)	1.12 (8)
**Dummy**	1.69 (2)	0.43 (6)	−1.00 (2)	−1.25 (1)
**Gedeone**	−0.42 (6)	−0.51 (3)	−0.64 (3)	1.08 (7)
**Gipsy**	−0.34 (5)	−1.37 (1)	−0.09 (5)	−0.92 (3)
**Indy**	−0.31 (4)	−1.27 (2)	−0.01 (6)	−0.74 (4)
**Minnie**	−0.80 (8)	1.20 (8)	1.81 (9)	−1.03 (2)
**Mousse**	−0.19 (3)	1.50 (9)	−1.13 (1)	0.39 (6)
**Nessie**	1.76 (1)	−0.19 (5)	1.34 (8)	1.17 (9)

## Supplementary Materials

The following are available online at https://www.mdpi.com/2076-2615/9/10/833/s1; Appendix A: The Ethogram Utilised in This Study for the Observational Sessions before, during and after the AAI Anxiety (Displacement Activities + Stereotyped Behaviours).
