# Supplementary material for: Evaluating Stress in Dogs Involved in Animal-Assisted Interventions
Source: Animals (Basel). 2019 Oct 19;9(10):833. doi: 10.3390/ani9100833 (PMC6827148; doi:10.3390/ani9100833)
Supplement: Supplementary file 1 [file animals-09-00833-s001.pdf]

Appendix A

The Ethogram Utilised in This Study for the Observational Sessions before, during and after the AAI Anxiety (Displacement Activities + Stereotyped Behaviours).

1.1. Displacement Activities

Scratching: raising one hind leg and vigorously scratching part of the body.

Muzzle licking: passing the tongue over the muzzle.

Yawning: opening the mouth and inhaling and exhaling air.

Auto-grooming: cleaning itself with the tongue and the teeth. Drooling: the dog flows saliva out of the mouth.

Panting: the dog breathes quickly and loudly through the mouth.

1.2. Stereotyped Behaviours

Repetitive pacing in circles: repetitive walking in a circle.

Licking or biting compulsively: repeatedly licking or biting the wall and/or objects.

Catching flies: trying to catch an imaginary fly with the mouth, clutching at empty air with the teeth.

Self-mutilation: licking itself continuously in same part of the body, so intensely to cause abrasions or even wounds.

1.3. Attention

Raising ears: the dog raises the ears because something catches his attention.

Looking at conductor: the dog turns his head and looks at the conductor.

Looking at patient: the dog turns his head and looks at the patient.

Looking at observer: the dog turns his head and looks at the observer.

Looking at other operator: the dog turns his head and looks at the operator.

Answer a command: the dog executes a command.

No-attention: the dog does not pay attention to people (voice or physical commands).

1.4. Olfactory Investigation

Sniffing environment: putting the muzzle on the ground, on the wall, or on the objects in the cage, the dog sniffs the environment.

Sniffing air: raising the head, moving the nostrils and breathing the air to perceive odours.

Sniffing human: pointing the muzzle towards the conductor/patient/observer/operator, the dog moves the nostrils clearly trying to perceive the odours of the observer.

1.5. Dominant Behaviour:

Raised tail: the tail is held high while it is still.

Wagging with the tail held high: moving the tail sideward while held high.

Pointer: holding the ears forwards while assuming an upright body posture with head and tail held high, legs straight and stiff.

Urinate with raised leg: the dog urinates raising one of its hind legs.

1.6. Aggressive Behaviour:

Growling: emitting a deep and long threat vocalisation.

Raising fur: raising the fur of the head, body and tail so that the dog appears to have a larger size and is thus more threatening.

Curling lip: the dog curls the higher lip.

Showing teeth: the dog curls both the lips to show teeth.

Sideways glance: the dog looks at someone not directly, in a very sinister way.

1.7. Submissive Behaviour:

Avoiding physical contact: the dog moves away when the someone tries to touch him.

Lowering head: lowering the head in front of the observer, another human or another dog.

Cringing: lying with the ventral region in contact with the ground.

Tail between the legs: holding the tail down or tightly between the hind legs and against the belly.

Lying down on back: laying down on the back exposing the ventral side of the chest and sometimes the abdomen.

Hiding behind the conductor: the dog goes to hide behind the conductor.

1.8. Vocal Communicability:

Barking: emitting an abrupt, loud, noisy, and often repetitive vocalisation characteristic of dogs.

Whining: emitting a mournful vocalisation.

Howling: emitting a vocalisation that consists of a long, high and mournful sound; characteristic of wolves, quite rare in dogs.

Snorting: emitting a vocalisation while puffing out its cheek and emitting air.

1.9. Affiliative Behaviour:

Waving tail: the tail is wagged sideward but not held high, in a relaxed manner.

Inviting to play: the dog tries to invite to play the conductor/patient/observer/operator, usually folding on the forelegs.

Giving the foreleg: raising one of the forelegs and leaning it in the direction of the observer.

Licking hands: licking the hand of the conductor/patient/observer/operator.

Requesting attention: the dog tries to catch the attention of someone, usually moving and barking.

1.10. Resting:

Sitting: sitting down with the rump leaning on the ground.

Lying: lying down on the ground.

Dozing: curling up, the dog is half asleep.

1.11. Playfulness Behaviour:

Inviting a human to play: the dog bends down with the forelegs outstretched on the ground and the rump upwards, or brings an object, runs around and jumps.

Playing: the dog play with the conductor/patient/observer/operator.
